# Early Reperfusion Hemodynamics Predict Recovery in Rat Hearts: A Potential Approach towards Evaluating Cardiac Grafts from Non-Heart-Beating Donors

**DOI:** 10.1371/journal.pone.0043642

**Published:** 2012-08-21

**Authors:** Monika Dornbierer, Mathieu Stadelmann, Joevin Sourdon, Brigitta Gahl, Stéphane Cook, Thierry P. Carrel, Hendrik T. Tevaearai, Sarah L. Longnus

**Affiliations:** 1 Department of Cardiovascular Surgery, Inselspital, Berne University Hospital and University of Berne, Berne, Switzerland; 2 Cardiology, University & Hospital, Fribourg, Switzerland; 3 University of Auvergne, Institute of Technology, Clermont-Ferrand, France; Sapienza University of Rome, Italy

## Abstract

**Aims:**

Cardiac grafts from non-heartbeating donors (NHBDs) could significantly increase organ availability and reduce waiting-list mortality. Reluctance to exploit hearts from NHBDs arises from obligatory delays in procurement leading to periods of warm ischemia and possible subsequent contractile dysfunction. Means for early prediction of graft suitability prior to transplantation are thus required for development of heart transplantation programs with NHBDs.

**Methods and Results:**

Hearts (n = 31) isolated from male Wistar rats were perfused with modified Krebs-Henseleit buffer aerobically for 20 min, followed by global, no-flow ischemia (32°C) for 30, 50, 55 or 60 min. Reperfusion was unloaded for 20 min, and then loaded, in working-mode, for 40 min. Left ventricular (LV) pressure was monitored using a micro-tip pressure catheter introduced via the mitral valve. Several hemodynamic parameters measured during early, unloaded reperfusion correlated significantly with LV work after 60 min reperfusion (*p*<0.001). Coronary flow and the production of lactate and lactate dehydrogenase (LDH) also correlated significantly with outcomes after 60 min reperfusion (*p*<0.05). Based on early reperfusion hemodynamic measures, a composite, weighted predictive parameter, incorporating heart rate (HR), developed pressure (DP) and end-diastolic pressure, was generated and evaluated against the HR-DP product after 60 min of reperfusion. Effective discriminating ability for this novel parameter was observed for four HR*DP cut-off values, particularly for ≥20 *10^3^ mmHg*beats*min^−1^ (*p*<0.01).

**Conclusion:**

Upon reperfusion of a NHBD heart, early evaluation, at the time of organ procurement, of cardiac hemodynamic parameters, as well as easily accessible markers of metabolism and necrosis seem to accurately predict subsequent contractile recovery and could thus potentially be of use in guiding the decision of accepting the ischemic heart for transplantation.

## Introduction

The discrepancy between supply and demand of donor organs is currently a major challenge in heart transplantation and is expected to worsen [1], [2]. In recent years, this gap between supply and demand has led to a renewed interest in non-heart-beating donors (NHBDs) as a source of potential cardiac grafts. In fact, NHBDs are already considered as an additional pool, alternative to brain-dead donors, for various organs including kidneys, livers, lungs and pancreatic islets. Although the first heart transplantation was performed with a NHBD graft in 1967 [3], the use of NHBDs hearts as a source of organs for cardiac transplantation was rapidly discontinued as clear criteria of brain death were defined shortly afterwards. Nevertheless, recent case reports have successfully reconfirmed the feasibility of orthotopic transplantation of NHBDs’ hearts in adult and pediatric patients [4], [5]. As a matter of fact, it was recently estimated that if hearts from NHBDs were to be used for transplantation, the donor supply could increase up to 17% in adults [6], [7] and 42% in pediatric patients [8].

Use of NHBDs in the clinical setting of cardiac transplantation, however, has not yet been adopted. This might be due, on one hand, to good results obtained with an optimal myocardial preservation strategy allowed by a coordinated organ procurement protocol after brain death; and, on the other hand, to the widespread belief that a period of warm ischemia always triggers irreversible myocardial injury [5] and subsequent post-operative contractile dysfunction. It is indeed true that NHBDs are legally required to undergo a “hands-off” period, which results in a period of warm ischemia of 2–10 min after cardiocirculatory arrest, during which cardioprotective interventions are limited for ethical reasons [9]. Several studies have clearly demonstrated, however, that a normal heart preserves sufficient integrity for transplantation if warm ischemia is limited to 20–30 min [10], [11], [12]. Therefore, a window of opportunity, albeit limited, does exist for useable cardiac grafts from NHBDs and should most probably be exploited, especially if reliable assessments could predict mid- and long-term functional prognosis. This could be even more advantageous if the window of opportunity could be widened. In this sense, we were able to demonstrate in a previous series of experiments, that functional recovery could be prolonged to 50–55 minutes providing a slight reduction in heart temperature (32°C) was initiated immediately after cardio-circulatory arrest (data not shown).

Heart evaluation is particularly important in the setting of NHBDs; given that from one donor to another, conditions of cardio-circulatory arrest and ischemic length and severity may differ considerably, thereby leading to variable degrees of tissue injury. Furthermore, not only ischemic injury, but also the chosen reperfusion strategy, will affect the prognosis of a NHBD’s heart. Consequently, and in order to consider this reperfusion injury, evaluation of a NHBD’s heart should be performed after reperfusion. Currently, only very few reports have proposed potential evaluation methods to predict recovery of cardiac contractile function applicable to NHBDs. These methods include measurement of coronary flow [13], [14], the use of an isolated myocardial perfusion system [15], [16] or fractional anisotropy [17], however, the latter two approaches require highly specialized equipment and considerable amounts of time.

In the current study, we aimed to simulate a NHBD setting in order to investigate, during a brief period of unloaded reperfusion, the potential of hemodynamic and biochemical markers that may predict recovery, and therefore eligibility, of NHBD hearts for cardiac transplantation. To do so, we chose to reduce the temperature of the graft after onset of cardiac arrest since, as previously mentioned, this might be a preliminary condition to reduce the damage of NHBD hearts. By using this approach, we also aimed to take advantage of a broader range of ischemic times, enabling us to more precisely define the degree of post-ischemic contractile recovery.

## Methods

### Materials

Bovine albumin (fraction V), palmitic acid and sodium-L-lactate were purchased from Sigma-Aldrich (Buchs, Switzerland) and insulin from Nordisk Pharma (Actrapid HM 100 IU/mL, Küsnacht, Switzerland). All other chemicals were obtained from Merck (Darmstadt, Germany). Dialysis membrane was purchased from Spectrum Labs (Rancho Dominguez, USA).

### Ethics Statement

All experimental procedures were performed in compliance with the European Convention for Animal Care and approved by the Swiss animal welfare authorities (Authorization Numbers: 58/08 and 11/11). All surgery was performed under anesthesia and all efforts were made to minimize suffering.

### Isolated Heart Preparation

The experimental set-up consisted of a modified isolated rat heart system as previously described [18]. Adult male Wistar rats (n = 31, weighing 515±53 g), fed on a standard laboratory diet *ad libitum*, were anesthetized intraperitoneally using ketamine 1.3 µL/g (Narketan, Vetoquinol AG, Bern) and xylazine 0.7 µL/g (Xylapan, Vetoquinol AG, Bern). Hearts were then rapidly excised and placed immediately in ice-cold, modified Krebs-Henseleit bicarbonate (KHB) buffer containing (mM): NaCl 118, KCl 4.7, KH_2_PO_4_ 1.2, MgSO_4_•7H_2_O 1.2, CaCl_2_ (dihydrate) 1.25, NaHCO_3_ 25 and glucose 5.5 (Buffer A). Hearts were weighed, aortae cannulated and retrograde/Langendorff perfusion (unloaded) was initiated with Buffer A equilibrated with 95% O_2_/5% CO_2_ at a pressure of 60 mmHg. Excess cardiac tissue was then removed, the pulmonary artery cut, and the left atria cannulated. Langendorff perfusion was then switched to loaded, aerobic, working-mode perfusion and a micro-tip pressure catheter (Millar instruments, Houston, Texas, USA) was forwarded into the left ventricle (LV) with access via the left atrial cannula.

### Experimental Protocol

**Figure 1:**
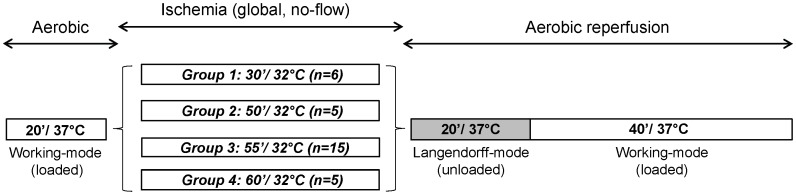
Perfusion protocol. Hearts (n = 31) were perfused for 20 min in aerobic working-mode, followed by global, no-flow ischemia. Four different ischemic periods at 32°C were investigated: 30 min (n = 6), 50 min (n = 5), 55 min (n = 15) and 60 min (n = 5). Hearts were then reperfused for 60 min, with the first 20 min in an unloaded-mode.

To establish baseline conditions, hearts were perfused for 20 min in the aerobic, working mode. The experimental protocol was then started only if LV developed pressure–heart rate (HR) product was ≥23 (mmHg*beats*min^−1^*10^−3^). Global, no-flow ischemia was initiated by clamping perfusate lines and placing the heart in a tissue bath filled with substrate-free Buffer A gassed with 95% N_2_/5% CO_2_ at 32°C. An ischemic temperature of 32°C was chosen as, at this temperature, increases in ischemic duration lead to a less rapid decline in post-ischemic recovery versus ischemia at 37°C, permitting thus the precise generation of a broader range of post-ischemic recoveries. Four different ischemic periods were investigated: 30 min (n = 6), 50 min (n = 5), 55 min (n = 15) and 60 min (n = 5). A reperfusion period of 60 minutes followed, with the initial 20 min in an unloaded-mode, with a constant aortic pressure of 60 mmHg (Figure 1).

The loaded, aerobic perfusion condition was characterized by an atrial filling pressure of 11.5 mmHg, an afterload-column set to 80 mmHg, with perfusion buffers maintained at 37°C and oxygenated with 95% O_2_/5% CO_2_. From the establishment of the working preparation, hearts were perfused only with recirculating KH buffer supplemented with 1.2 mM palmitate, 3% albumin; this includes the entire period of reperfusion, both unloaded and loaded. More specifically, this buffer contained: NaCl 118 mM, KCl 4.7 mM, KH_2_PO_4_ 1.2 mM, MgSO_4_•7H_2_O 1.2 mM, CaCl_2_ 1.25 mM, NaHCO_3_ 25 mM, glucose 5.5 mM, insulin 100 µIU/mL, albumin 3%, lactate 0.5 mM and palmitic acid 1.2 mM (Buffer B); concentrations representing physiologic levels of glucose, insulin and lactate and high levels of fatty acids. High palmitate levels were chosen as circulating fatty acids are typically elevated in humans undergoing cardiac surgery [19], and are known to be detrimental to recovery of contractile function following ischemia [20].

LV pressure curves and heart rate were continuously recorded using a micro-tip pressure catheter (Millar instruments, Houston, Tex) coupled to a high performance data acquisition system (PowerLab, ADInstruments, Spechbach, Germany). Pressures were analyzed at 10 and 20 min of aerobic perfusion and at 5, 10, 15, 20, 30, 40 and 60 min reperfusion. In order to reduce variations in baseline drift associated with the micro-tip pressure catheter, mean pre-ischemic left ventricular minimum pressure was set to zero and subsequent pressure measurements were adjusted correspondingly.

Coronary flow (CF) and aortic flow (AF) were measured by timed collection at above-mentioned time-points (10 and 20 min of pre-ischemic perfusion and at 5, 10, 15, 20, 30, 40 and 60 min reperfusion). Samples of circulating buffer and coronary effluent for quantification of markers of metabolism and necrosis were also taken at these time points. Additional buffer samples for baseline perfusate measurements were collected at the beginning of the pre-ischemic working-mode and at the end of ischemia, immediately before the initiation of reperfusion.

#### Hemodynamics

The following hemodynamic parameters were assessed:

- heart rate (HR [beats*min^−1^]),
- peak systolic pressure (PSP [mmHg]),
- LV minimum pressure (P_min_ [mmHg]),
- LV end-diastolic pressure (EDP [mmHg]),
- LV developed pressure (DP [PSP-P_min_; mmHg]),
- maximum and minimum first derivatives of LV pressure (dP/dt_max_ and dP/dt_min_ [mmHg*s^−1^]),
- coronary flow (CF [mL*min^−1^]),
- cardiac output (CO [CF+AF; mL*min^−1^]), and
- LV work
  1. PSP-HR product [mmHg*beats*min^−1^],
  2. DP-HR product [mmHg*beats*min^−1^],
  3. triple product-PSP (TP(PSP) [PSP*dP/dt_max_*HR; mmHg^2^*beats*min^−1^*s^−1^]
  4. triple product-DP (TP(DP) [DP*dP/dt_max_ *HR; mmHg^2^*beats*min^−1^*s^−1^]).

#### Markers of metabolism and necrosis

Lactate levels were measured in buffer samples with an iSTAT analyzer (Abbott, Baar, Switzerland) using CG4+ cartridges (Axonlab, Baden, Switzerland). Lactate dehydrogenase (LDH) was assessed with a Roche MODULAR P800 analyzer (Roche Diagnostics Corp, Indianapolis, USA).

#### Predictive parameters

Predictive parameters were assessed at 5 and 10 min reperfusion. Absolute and relative differences between 5- and 10- min values were also analyzed. Predictive parameters included all hemodynamic parameters mentioned above, with the exception of cardiac output, as well as release of lactate and lactate dehydrogenase.

#### Outcome parameters

Outcomes were measured at 60 min reperfusion, and are presented as absolute value or as percent of mean pre-ischemic value. Outcome parameters included all hemodynamic parameters mentioned above.

### Data Analysis

All values are expressed as mean±SD unless stated otherwise. Data were analyzed using SPSS for Windows (version 17.0, SPSS Inc, Chicago, Ill). Statistical analyses were performed using an ANOVA with repeated measures for an overview of interactions between ischemic group and measured parameters at baseline or 60 min reperfusion. Where significant overall results were observed, comparisons between groups of particular interest were performed using t-tests. Associations between outcome parameters measured after 60 min reperfusion and predictive parameters at 5 and 10 min reperfusion were investigated using Spearman correlations. Multiple linear regression analysis was used to generate a composite, weighted predictive parameter based on intraventricular pressure measurements. The predictive quality of this composite parameter was examined using ROC analysis. All *p*-values were two-sided, adjusted for multiple comparisons, and reported after correction. Corrected *p*-values <0.05 were considered statistically significant.

## Results

### Baseline Parameters

Thirty-one hearts were included in the study. Average pre-ischemic characteristics of hearts are shown in Table S1. No significant difference was observed between ischemic groups, with the following exceptions: heart weight was significantly greater in the 55-min and 60-min ischemic groups compared with the 30- min ischemic group (*p* = 0.033 and *p* = 0.015, respectively); dP/dt_max_ was significantly lower in the 55-min ischemic group compared with the 30- min ischemic group (*p* = 0.015) and TP(PSP)/TP(DP) was significantly lower in the 55-min ischemic group compared with the 30- min ischemic group (*p* = 0.015).

### Post-ischemic Cardiac Function

**Figure 2:**
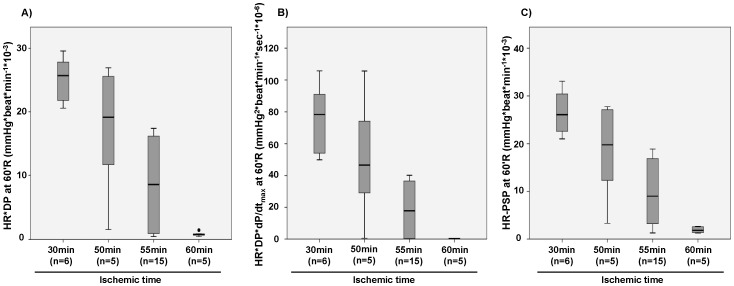
Post-ischemic contractile recovery. Functional recovery at 60 min reperfusion in hearts subjected to various periods of ischemia at 32°C. **A**) Heart rate (HR) - developed pressure (DP) product **B**) HR - DP - dP/dt_max_ product **C**) HR - peak systolic pressure product.

Hemodynamic function measured 60 min after reperfusion was progressively reduced with increasing ischemic times (Figure 2). Importantly, when considering all ischemic groups together after 60 min reperfusion, hemodynamic data, expressed as percentage of pre-ischemic values, covered almost the entire range of functional recovery. For instance, HR*PSP and HR*DP values ranged from approximately 5–90% of pre-ischemic measurements, while a range of approximately 0.5–60% recovery was observed for HR*PSP*dP/dt_max_ and HR*DP*dP/dt_max_ (Table 1).

**Table 1:** Hemodynamic parameters after 60 minutes reperfusion.

	Ischemic group								
	30 minutes (n = 6)		50 minutes (n = 5)		55 minutes (n = 15)			60 minutes (n = 5)	
	%	*Abs 60’R*	%	*Abs 60’R*	%	*Abs 60’R*	%		*Abs 60’R*
HR [beats*min^−1^]	118±24	*264±24*	88±24	*204±55*	66±24^b^	*163±62* b	40±23^a^		*100±57* a
**PSP [mmHg]**	78±9.9	*100±9*	68±29	*85±39*	49±26	*57±32*	22±13^b^		*24±14* b,c
**P_min_ [mmHg]**	–	*5.0±7.4*	–	*5.4±2.9*	–	*7.1±4.6*	–		*14.0±10*
**EDP [mmHg]**	166±126	*11±8*	173±43	*11±2*	188±74	*12±4*	253±204		*16±10*
**DP [mmHg]**	74±8	*95±5.5*	64±31	*80±42*	43±29	*50±36*	9.2±4.0b,c		*10±4.3* b,c
dP/dt_max_ [mmHg*s^−1^]	68±17	*2971±457*	63±31	*2417±1306*	45±29	*1475±963*	10±5a,c		*322±137* b,c
dP/dt_min_ [mmHg*s^−1^]	73±8.7	*−2162±220*	54±28	*−1798±966*	36±24^a^	*−1106±751*	10±4.3b,c		*−282±113* a,c
HR*PSP [mmHg*beats*min^−1^*10^−3^]	91±16	*26.5±4.6*	63±35	*18.0±10*	34±23^b^	*9.93±6.9* b	6.8±2.2b,d		*1.93±0.65* b,c
HR*DP [mmHg*beats*min^−1^*10^−3^]	86±14	*25.2±3.7*	59±36	*17.0±10.5*	31±23^b^	*8.86±7.0* b	3.0±1.4b,d		*0.854±0.38* b,d
TP (PSP) [mmHg^2^*beats*min^−1^*s^−1^*10^−6^]	62±18	*80.4±25*	47±32	*53.5±42*	21±18^b^	*20.1±18* b	0.6±0.2b,c		*0.564±0.13* b,c
TP (DP) [mmHg^2^*beats*min^−1^*s^−1^*10^−6^]	59±16	*76.2±22*	46±31	*51.2±40*	20±17^b^	*18.8±17* b	0.3±0.1b,c		*0.255±0.11* b,c
Cardiac output [mL*min^−1^]	52±19	*23±7*	48±32	*30±20*	24±18	*12±10*	12±6^a^		*6.0±3.1* c
Coronary flow [mL*min^−1^]	75±24	*14±4*	81±51	*24±17*	50±39	*12±10*	21±11		*6.0±3.1*

Data expressed as mean±SD.

a *p*<0.05 vs 30-minute ischemic value;

b *p*<0.01 vs 30-minute ischemic value;

c *p*<0.05 vs 50-minute ischemic value;

d *p*<0.01 vs 50-minute ischemic value;

DP: Developed pressure; EDP: LV end-diastolic pressure; HR: Heart rate; P_min_: Minimum LV pressure; PSP: Peak systolic pressure; TP (DP): Triple product (DP); TP (PSP): Triple product (PSP).

### Early Reperfusion Predictive Parameters

**Figure 3:**
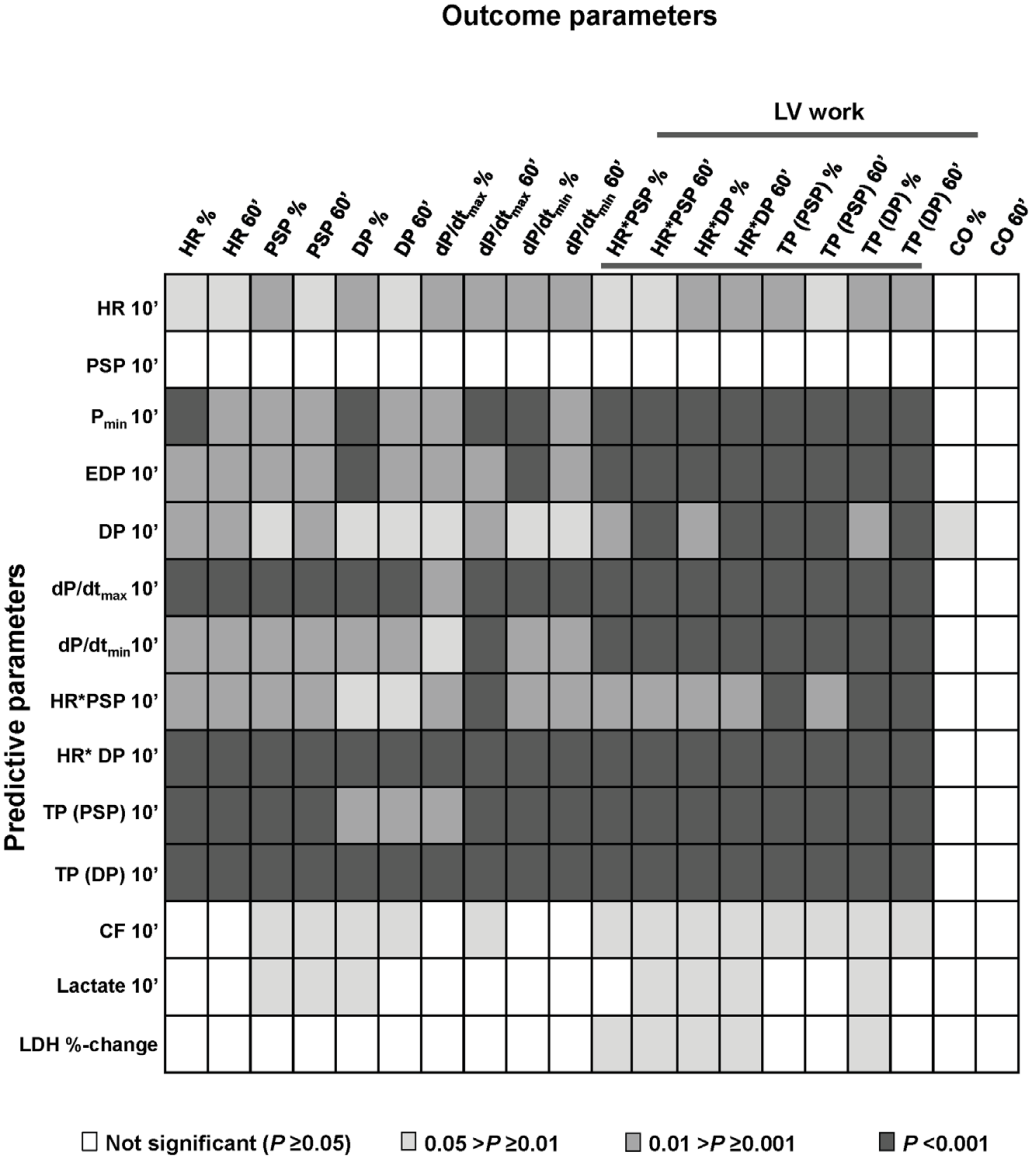
Spearman correlations between predictive and outcome parameters. All predictive parameters were measured at 10-min reperfusion except for LDH, which was calculated as percent change between 5- and 10-min reperfusion. Outcome parameters were measured at 60-min reperfusion and either taken as absolute value (60’) or percentage of mean pre-ischemic value (%).

For several parameters assessed at 5 and/or 10 min of unloaded reperfusion, significant correlations were found with functional outcome parameters recorded during the subsequent loaded reperfusion phase (60 min reperfusion). A greater number of early reperfusion parameters were found to correlate with outcomes when assessed at 10 min versus 5 min or versus 5- to 10-min absolute and relative changes, with the exception of LDH release (data not shown). As such, apart from LDH release, early reperfusion parameters are presented for the 10-min reperfusion time-point only. Examples of correlations between hemodynamic predictive parameters, and the outcome of HR*DP at 60-min reperfusion are presented in Figure S1. An overview of the correlations between “predictive” and “outcome” parameters is given in Figure 3. Predictive parameters of particular interest are presented in more detail in Table 2.

**Table 2:** Rho values for statistically significant predictor-outcome correlations^a^.

	Outcome parameters [*Rho*]							
Predictive Parameters	HR*PSP	HR*PSP	HR*DP	HR*DP	TP (DP)	TP (DP)	TP (DP)	TP (DP)
	(%)	(60’ R)	(%)	(60’)	(%)	(60’R)	(%)	(60’R)
**Heart rate 10’**	0.507	0.485	0.554	0.539	0.568	0.502	0.543	0.500
**P_min_ 10’**	−0.660	−0.635	−0.729	−0.688	−0.653	−0.656	−0.681	−0.675
**EDP 10’**	−0.644	−0.614	−0.710	−0.663	−0.642	−0.638	−0.670	−0.659
**DP 10’**	0.597	0.617	0.603	0.624	0.607	0.635	0.593	0.623
**dP/dt_max_ 10’**	0.718	0.728	0.732	0.732	0.743	0.744	0.733	0.749
**dP/dt_min_ 10’**	−0.606	−0.627	−0.621	−0.636	−0.650	−0.677	−0.630	−0.681
HR*PSP 10’	0.580	0.580	0.585	0.596	0.625	0.592	0.618	0.618
HR*DP 10’	0.748	0.747	0.767	0.773	0.792	0.759	0.787	0.784
**TP (PSP) 10’**	0.695	0.698	0.703	0.713	0.739	0.718	0.728	0.738
**TP (DP) 10’**	0.729	0.733	0.753	0.757	0.771	0.751	0.767	0.771
**Coronary flow 10’**	0.471	0.470	0.458	0.463	0.500	0.496	0.496	0.502
**Lactate production 10’**	NS	−0.583	−0.632	−0.605	−0.620	−0.559	−0.615	−0.576
**LDH release %-change**	−0.608	−0.591	−0.623	−0.586	−0.605	−0.554	−0.598	NS

Data are expressed as *Rho*. *Rho*: Spearman’s rank correlation coefficient;

a All Rho values correspond to statistically significant correlations, for *p*-values see Figure 3; Outcome parameters: %: 60-min reperfusion value as percent of mean pre-ischemic values; 60’R: value measured at 60-min reperfusion; Predictive parameters: 10’: Measurement at 10-min reperfusion; %-change: percentage change between 5- and 10-min reperfusion values; DP: developed pressure; EDP: LV end-diastolic pressure; HR: heart rate; LDH: Lactate dehydrogenase; NS: Not significant; P_min_: LV minimum pressure; PSP: LV peak systolic pressure; TP (DP): triple product (DP) – HR*DP*dP/dt_max_; TP (PSP): triple product (PSP) – HR*PSP*dP/dt_max._

#### Hemodynamics

Predictive parameters of dP/dt_max_, dP/dt_min_, P_min_, EDP and LV work (measured as HR*DP, TP(PSP) and TP(DP)), all assessed after 10 min of unloaded reperfusion, strongly correlated (*p*<0.001) with all eight LV work outcome parameters (evaluated at 60 min reperfusion and expressed as percent pre-ischemic and absolute values; Figure 3). Of particular note, all Rho values for correlations with the eight LV work outcome parameters were greater than 0.7 for the predictors HR*DP, TP(DP) and dP/dt_max_; ranging from 0.72 to 0.79 (Table 2).

#### Coronary perfusion

ENREF_28CF measured at 10 min reperfusion correlated with several outcomes, including all eight measures of LV work, with Rho values ranging between 0.46 and 0.50 (*p*<0.05; Table 2 and Figure 3).

#### Markers of metabolism and necrosis

Lactate production was not detectable in any of the hearts during the initial 20 min of pre-ischemic, aerobic perfusion (data not shown). During reperfusion, however, a measurable increase in lactate production was observed in the circulating buffer and in the coronary effluent of all hearts. A negative correlation was found between lactate production assessed at 10 min reperfusion and four outcome measures of LV work at 60 min reperfusion (HR*PSP – absolute value, HR*DP– percentage pre-ischemic and absolute values, and TP(DP) – percentage pre-ischemic value); with Rho values between −0.56 and −0.63 (Table 2 and Figure 3).

The percentage change of LDH released between 5 and 10 min reperfusion was negatively correlated with five outcome measures of LV work at 60 min reperfusion (HR*PSP – percentage pre-ischemic and absolute values, HR*DP – percentage pre-ischemic and absolute values, and TP(DP) – percentage pre-ischemic value); with Rho values between −0.55 and −0.62 (Table 2 and Figure 3).

#### Predictive quality

Multiple, linear regression analysis was used to generate a weighted, composite predictor using predictive parameters EDP and HR*DP and absolute 60-min reperfusion HR*DP values as outcome (choice of predictive parameters based on Spearman correlation analysis and on the logical complementarity of parameters; the former representing LV compliance, and the latter representing LV work). The following relationship was obtained:

Estimated HR*DP_(60’ reperfusion)_  = 1.082*(HR*DP_(10’ reperfusion)_) −320.114*(EDP_(10’ reperfusion)_) +8.245*10^3^.

**Figure 4:**
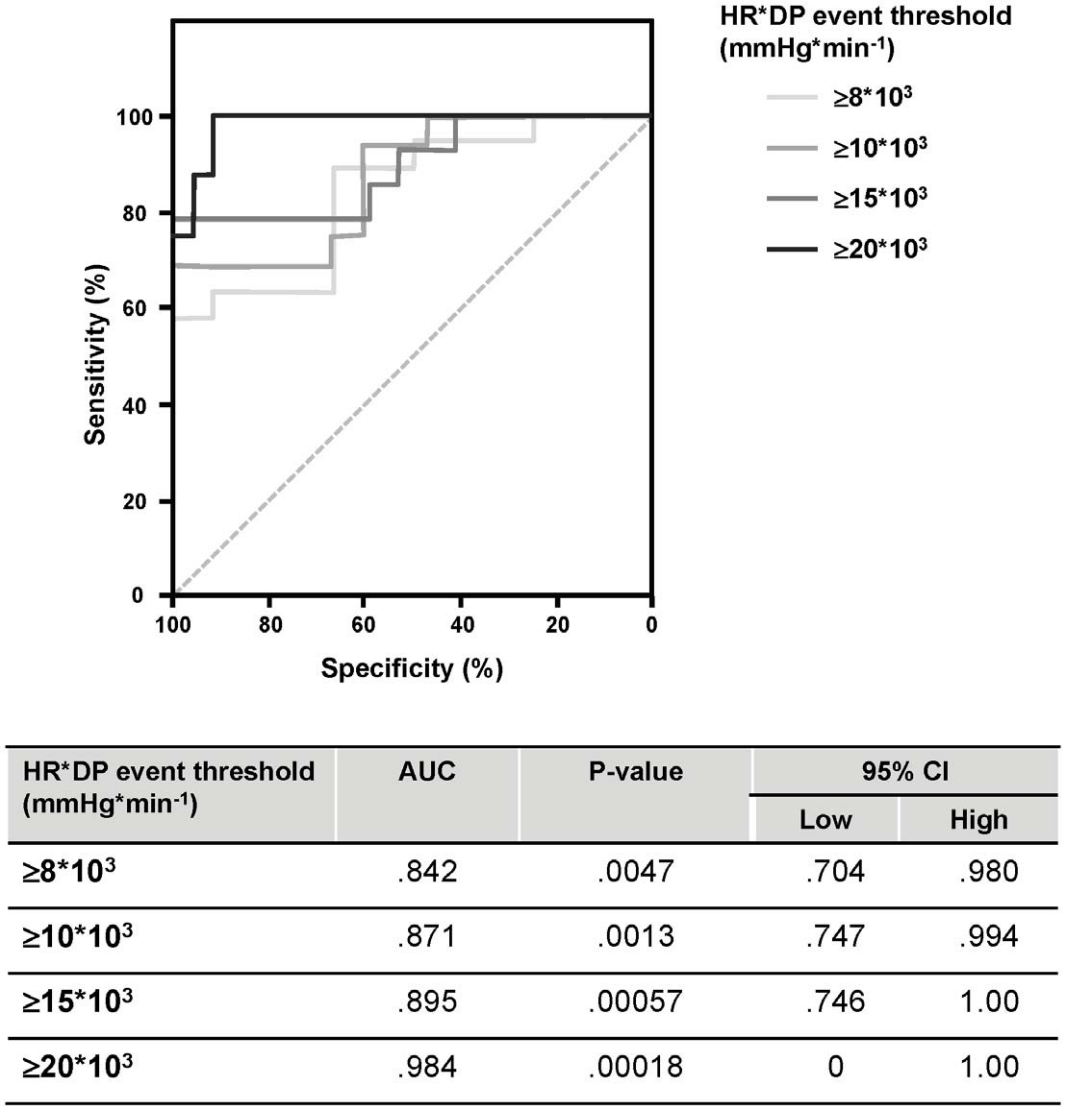
ROC analysis for composite predictive parameter. ROC analysis for a novel, composite hemodynamic-based predictive parameter of functional recovery after ischemia based on early reperfusion measures of HR-DP product and EDP. Discriminating ability of this composite predictive parameter was evaluated using absolute values of HR*DP (mmHg*beats*min^−1^) with the following event threshold cut-off values: ≥8*10^3^, ≥10*10^3^, ≥15*10^3^, and ≥20 *10^3^.

This composite predictor was then evaluated using ROC analysis using the following event threshold 60-minute HR*DP cut-off values: ≥8*10^3^; ≥10*10^3^; ≥15*10^3^; and ≥20 *10^3^ mmHg*beats*min^−1^ (Figure 4). Effective discriminating ability was observed for all event cut-off values, and particularly for the threshold of ≥20 *10^3^ mmHg*beats*min^−1^.

## Discussion

**Figure 5:**
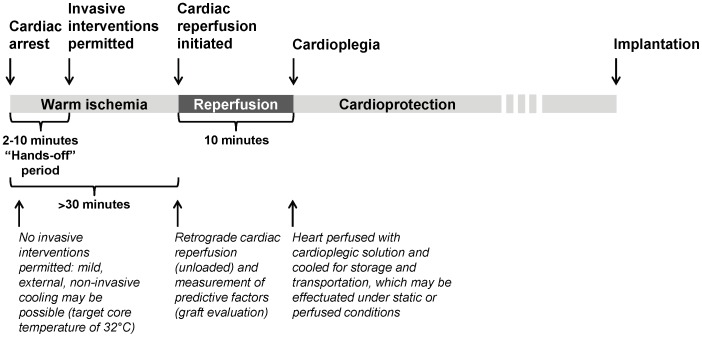
Schematic representation of NHBD-heart procurement/evaluation/transplantation process in a potential clinical scenario. Following a precipitating event (e.g. trauma accident, removal of life-support) leading to cardio-circulatory arrest, the heart is subjected to a variable period of warm ischemia and an obligatory hands-off period. Once invasive interventions are permitted, we propose that the heart could first be reperfused *in situ* or *ex vivo* in an unloaded manner for 10 minutes, during which time predictive parameters could be measured for graft evaluation. Immediately following this 10-minute period, and in the event that evaluation predicts a positive outcome, cardioplegia could be commenced and the heart prepared for transport.

In the present study, we have identified hemodynamic and biochemical parameters that appear highly predictive regarding the chances of myocardial contractile recovery after prolonged ischemia. Most importantly, the correlation with functional recovery could be established almost immediately after a simple brief period of unloaded retrograde *ex-vivo* reperfusion. This approach has the potential to be particularly useful in a clinical setting of heart transplantation from NHBDs, as these measurements can be obtained rapidly (10 minutes after end of ischemia), are not technically challenging, and require little specialized equipment. Furthermore, in the context of NHBDs, most traditionally used means to assess suitability of the graft for transplantation, such as echocardiography and/or coronary angiography, may not be applicable. Our approach may therefore serve as a first step towards the development of a clinical protocol to evaluate the extent of warm ischemic damage and, consequently, to reliably predict the prognosis once the graft is transplanted and reperfused (Figure 5).

Given that NHBDs are generally not considered as a source of donor organs for cardiac transplantation due to tissue damage caused by variable warm periods of ischemia [4], the importance of a safe and reliable technique to test for heart eligibility is evident. Ideally, methods to evaluate suitable donor hearts should be easily and rapidly performed, and in accordance with ethical and cultural concerns in the context of cardiac transplantation from NHBDs. As such, we propose the assessment of heart suitability for transplantation with use of a retrograde coronary perfusion, immediately following the obligatory hands-off period at the time of procurement. This type of perfusion approach is easy to establish in either an *ex vivo* or an *in situ* configuration, and allows immediate evaluation of the graft. In the case of a result predicting suitability for transplantation, administration of cardioprotective agents for cardioplegic storage, organ transport and transplantation can be performed immediately thereafter.

This approach is not necessarily limited to NHBDs; it could potentially serve as a useful tool to evaluate function of any cardiac graft prior to transplantation. In fact, not only hearts from NHBDs are exposed to a damaging environment, i.e. warm ischemia and associated consequences. Indeed, hearts from brain-dead donors are also often subjected to hemodynamic instability. This hemodynamic instability may lead to cardiac damage and/or require pharmacological management that subsequently precludes heart donation [21], [22]. Therefore, our protocol could also be used towards a procedure for heart screening such that other marginal donors may be thoroughly evaluated, potentially further increasing donor organ availability.

In this study, we took advantage of a recently developed approach to produce a series of hearts with a broad range of hemodynamic function following ischemia and reperfusion. More specifically, in a previous series of experiments, we were able to demonstrate that a slight reduction of the heart temperature (32°C), initiated immediately after cardio-circulatory arrest, was able to prolong ischemic tolerance (data not shown). Following ischemia at 32°C, hearts recovered substantial hemodynamic function after 50–55 minutes, whereas functional recovery was completely abolished after 30 minutes ischemia at 37°C. For the current study we used a similar approach, which enabled us to employ a broader range of ischemic times, permitting a more precisely defined degree of post-ischemic contractile recovery.

In our experimental setting, we found that early reperfusion measures of EDP, P_min_, dP/dt_max_, dP/dt_min_ and LV work (HR*DP, TP(PSP) and TP(DP)) were more strongly correlated with outcome parameters compared with other hemodynamic parameters, biochemical markers or coronary flow. Interestingly, previously recognized parameters of cardiac recovery, such as lactate release [23] and coronary flow [13], [14] were less strongly correlated, and correlated with fewer outcomes compared with hemodynamic parameters in our model. Given these findings, we generated a composite, weighted predictor using HR*DP and EDP; parameters chosen for their strong correlations with LV work outcomes and their complementarity (the former representing LV work, and the latter representing LV compliance). Our novel predictive parameter provides statistically significant predictive value for discriminating amongst subsequent LV work outcomes and thereby offers a promising approach towards human cardiac graft evaluation prior to transplantation. Despite this fact, our work has been performed exclusively in a rat model and further investigation is clearly required before transposing this composite predictor to a clinical setting. Nevertheless, we provide evidence indicating that strong correlations can be established between early, accurately measurable parameters and functional, clinically relevant outcomes.

Indeed, in future studies, it will be critical to investigate the importance of including additional complementary parameters, such as biochemical measures, in graft evaluation. For example, a multi-step protocol of heart evaluation could be developed, with an initial estimation of graft suitability using hemodynamic measures, followed by the integration of complementary parameters such as laboratory data in order to obtain a reliable, robust and sensitive means of evaluation. Importantly, given that graft evaluation is likely to require a “go” versus “no-go” decision, only predictive parameters that can provide immediate results should be considered. In addition, since organ procurement from NHBDs cannot necessarily be anticipated, predictive parameters that require highly specialized equipment or expertise should be avoided, such that they can be measured easily and rapidly in most centers. Furthermore, utility of predictive the parameters described here should be validated in large animal transplantation models, and potentially in *ex vivo* human heart preparations, prior to the commencement of clinical trials.

Our study identifies a novel approach for evaluation of the suitability of NHBD hearts for transplantation, providing a promising tool for the development of a clinical protocol for the use of NHBDs. However, many aspects including precise procurement and donor treatment protocols, definition of cardiac death and hands-off time, acceptability limits for warm ischemia, identification of protective interventions, as well as legal and ethical issues must be addressed before widespread adoption of cardiac transplantation from NHBDs is likely to occur. Importantly, given that waiting-list mortality remains excessive, approximately 17% in patients in the US [6], [24], approaches directed towards the use of hearts from NHBDs clearly warrant further investigation.

### Limitations

We consider this work to be an early step towards the routine use of cardiac grafts from NHBDs, given that additional work is required for the translation of this approach to the clinic. As mentioned above, the predictive parameters described must be validated in a larger heart model, as our experiments were performed exclusively in rat hearts. Secondly, hearts were perfused in the absence of blood, but with physiologic buffer. As such, blood-associated events, such as modifications in inflammatory processes or reactive oxygen species scavenging, that occur during ischemia and reperfusion could not be taken into account. Nevertheless, given that circulating fatty acid levels are typically elevated in humans undergoing cardiac surgery [19], and are known to be detrimental to recovery of contractile function following ischemia [20], we perfused hearts in the presence of high palmitate levels to better approximate the clinical situation. Thirdly, it is possible that the use of ketamine/xylasine altered baseline, pre-ischemic hemodynamic function and consequently affected values for percentage recovery. However, we believe that ketamine/xylasine was a good anesthetic choice for this study, as we wished to avoid ischemic pre-conditioning effects caused by alternative anesthetics, such as volatile anesthetics or pentobarbital (severe respiratory depression). Finally, recovery after ischemia was limited to a period of 60 minutes. Therefore, the validity of our predictive parameters remains to be investigated with subacute and long-term recovery, as well as following cardioplegic storage.

## Supporting Information

Figure S1 **Associations between predictive and outcome parameters.** Example associations between hemodynamic parameters during early unloaded reperfusion and functional outcome after 60 minutes reperfusion. Heart rate-developed pressure product measured after 60 minutes reperfusion positively correlated with **A**) dP/dt_max_, **B**) Heart rate - developed pressure product and **C**) Heart rate - developed pressure - dP/dt_max_ product, all measured at 10 minutes reperfusion.
(TIFF)

Table S1 **Baseline (pre-ischemic) characteristics.** Data expressed as mean±SD. *p<0.05 vs. 30 min ischemia group; DP: Developed pressure; EDP: LV end-diastolic pressure; HR: Heart rate; PSP: Peak systolic pressure; TP (DP): Triple product (DP); TP (PSP): Triple product (PSP)
(DOC)
