# Supplementary figures and images for: Early Reperfusion Hemodynamics Predict Recovery in Rat Hearts: A Potential Approach towards Evaluating Cardiac Grafts from Non-Heart-Beating Donors
Source: PLoS One. 2012 Aug 21;7(8):e43642. doi: 10.1371/journal.pone.0043642 (PMC3424125; doi:10.1371/journal.pone.0043642)

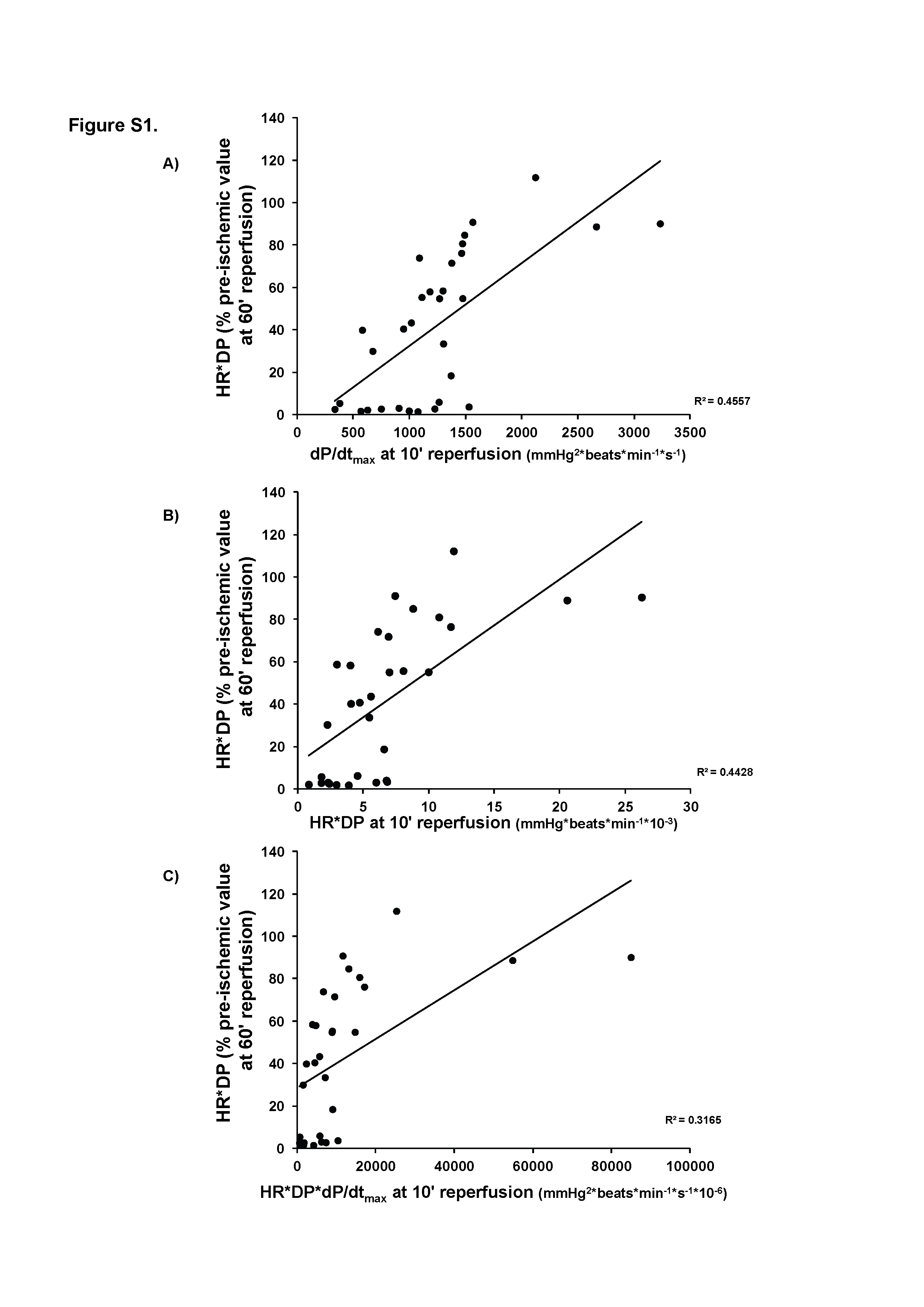

Supplement: Figure S1 — Associations between predictive and outcome parameters. Example associations between hemodynamic parameters during early unloaded reperfusion and functional outcome after 60 minutes reperfusion. Heart rate-developed pressure product measured after 60 minutes reperfusion positively correlated with A) dP/dtmax, B) Heart rate - developed pressure product and C) Heart rate - developed pressure - dP/dtmax product, all measured at 10 minutes reperfusion. (TIFF) [file pone.0043642.s001.tiff]
