# Supplementary material for: Early Reperfusion Hemodynamics Predict Recovery in Rat Hearts: A Potential Approach towards Evaluating Cardiac Grafts from Non-Heart-Beating Donors
Source: PLoS One. 2012 Aug 21;7(8):e43642. doi: 10.1371/journal.pone.0043642 (PMC3424125; doi:10.1371/journal.pone.0043642)
Supplement: Table S1 — Baseline (pre-ischemic) characteristics. Data expressed as mean±SD. *p<0.05 vs. 30 min ischemia group; DP: Developed pressure; EDP: LV end-diastolic pressure; HR: Heart rate; PSP: Peak systolic pressure; TP (DP): Triple product (DP); TP (PSP): Triple product (PSP) (DOC) [file pone.0043642.s002.doc]

**Table S1. Baseline (pre-ischemic) characteristics**

|  |  | | | **Ischemic groups** | | | |
| --- | --- | --- | --- | --- | --- | --- | --- |
|  | **All hearts** | | | **30 minutes** | **50 minutes** | **55 minutes** | **60 minutes** |
| **Number of hearts [n]** | | 31 | 6 | | 5 | 15 | 5 |
| **Heart weight [g]** | | 2.1±0.3 | 1.8±0.2 | | 1.9±0.2 | 2.2±0.3* | 2.4±0.1* |
| **HR [beats*min^-1^]** | | 242±22 | 229±35 | | 232±11 | 246±17 | 255±20 |
| **PSP/ DP [mmHg]** | | 119±12 | 129±14 | | 122±12 | 116±10 | 112±3 |
| **EDP [mmHg]** | | 6.7±1.3 | 7.3±2.0 | | 6.7±0.8 | 6.3±1.0 | 7.1±1.4 |
| **dP/dt_max_ [mmHg*s^-1^]** | | 3617±876 | 4597±1376 | | 3864±544 | 3277±501* | 3211±225 |
| **dP/dt_min_ [mmHg*s^-1^]** | | -3078±519 | -2978±290 | | -3274±473 | -3150±649 | -2785±162 |
| **HR*PSP/ HR*DP [mmHg*beats*min^-1^*10^-3^]** | | 29±2.1 | 29±2.2 | | 28±1.9 | 28±2.3* | 28±2.2 |
| **TP (PSP)/ TP(DP) [mmHg^2^*beats*min^-1^*s^-1^*10^-6^]** | | 104±26 | 133±33 | | 110±20 | 94±20 | 91±7 |
| **Cardiac output [mL*min^-1^]** | | 52±9 | 47±13 | | 61±3xx | 53±8 | 50±3 |
| **Coronary flow [mL*min^-1^]** | | 25±6 | 20±7 | | 31±6 | 24±3 | 29±5 |
| Data expressed as mean±SD. **p*<0.05 vs. 30 min ischemia group; DP: Developed pressure; EDP: LV end-diastolic pressure; HR: Heart rate; PSP: Peak systolic pressure; TP (DP): Triple product (DP); TP (PSP): Triple product (PSP) | | | | | | | |
